# Alterations of hepatic lipid content following COVID-19 in persons with type 2 diabetes

**DOI:** 10.1136/bmjdrc-2024-004727

**Published:** 2025-02-18

**Authors:** Yuliya Kupriyanova, Iryna Yurchenko, Pavel Bobrov, Frederik Bartels, Stefan Wierichs, Marc Jonuscheit, Benedict Korzekwa, Katsiaryna Prystupa, Martin Schön, Dania Mendez, Sandra Trenkamp, Volker Burkart, Robert Wagner, Vera Schrauwen-Hinderling, Michael Roden, The German Diabetes Study (GDS) Group, M Roden

**Affiliations:** 1 Institute for Clinical Diabetology, German Diabetes Center, Leibniz Center for Diabetes Research at Heinrich Heine University Düsseldorf, Düsseldorf, Germany; 2 German Center for Diabetes Research (DZD), Partner Düsseldorf, Neuherberg, Germany; 3 Institute for Biometrics and Epidemiology, German Diabetes Center, Leibniz Center for Diabetes Research at Heinrich Heine University Düsseldorf, Düsseldorf, Germany; 4 Department of Endocrinology and Diabetology, Medical Faculty and University Hospital Düsseldorf, Heinrich Heine University Düsseldorf, Düsseldorf, Germany; 5 Department of Radiology and Nuclear Medicine, Maastricht University Medical Center, Maastricht, Netherlands

**Keywords:** COVID-19, Diabetes Mellitus, Type 2

## Abstract

**Introduction:**

The study aimed to assess the effect of COVID-19 on hepatic lipid (HL) content, fibrosis risk, and adiposity in persons with type 2 diabetes.

**Research design and methods:**

Participants with type 2 diabetes with a history of mild COVID-19 (n=15, age 58±12 years, body mass index 30.9±5.2 kg/m^2^) were examined before (baseline) and 1 year (12±2 months) after (follow-up) recovery from COVID-19. Investigations for changes in metabolic risk comprised clinical examination, fasting blood sampling and MR-based measurements. Potential changes were corrected with the time course of the respective parameters in a group of participants who did not contract COVID-19 over the same time course (n=14, 61±6 years, 30.0±4.6 kg/m^2^).

**Results:**

COVID-19 resulted in a relative increase in HL content of 56% (95% CI 18%, 106%; p=0.04) measured as proton density fat fraction (HL-PDFF), corrected for the time course in the absence of COVID-19. While no changes in hepatic stiffness and volume, intramyocellular lipids, whole-body, subcutaneous and visceral adipose tissue volumes as well as homeostatic model assessment of insulin resistance and beta-cell function were observed.

**Conclusions:**

History of COVID-19 in persons with type 2 diabetes is associated with higher HL-PDFF after 1 year following recovery from infection.

**Trial registration number:**

NCT01055093.

**WHAT IS ALREADY KNOWN ON THIS TOPIC**

- It is known that COVID-19 can affect respiratory system and other organs, even after a relatively mild acute illness with unclear effects in the long-term.

**WHAT THIS STUDY ADDS**

- The study assessed the effect of COVID-19 on hepatic lipid content, fibrosis risk, and adiposity in persons with type 2 diabetes.

**HOW THIS STUDY MIGHT AFFECT RESEARCH, PRACTICE OR POLICY**

- Results demonstrate that even non-severe COVID-19 may exert a long-term effect on hepatic lipid content in persons with type 2 diabetes. A history of COVID-19 may increase the long-term risk for metabolic disorders.

## Introduction

 In addition to chronic effects on the respiratory system,^1^ COVID-19 can affect other organs, even after a relatively mild acute illness with unclear effects in the long-term.^2^ The post-COVID-19 syndrome may impact on body composition, particularly visceral adipose tissue (VAT),^3^ induce whole-body insulin resistance^4^ and maybe cause or worsen hepatic steatosis.^4^,^6^ These alterations could be specifically deleterious for persons with type 2 diabetes, which per se associates with a higher risk for metabolic dysfunction-associated steatotic liver disease (MASLD) and its advanced form, hepatic fibrosis. One can hypothesize that infection with SARS-CoV-2 leading to COVID-19 thereby accelerates MASLD progression in type 2 diabetes. The mechanism of whether and how COVID-19 impacts on the liver is yet unknown but could include direct viral damage and/or other virus-induced mechanisms, such as immune dysregulation, inflammation and hypoxic/ischemic injury.^4 7^

To date, effects of COVID-19 on hepatic lipid (HL) content and adipose tissue compartments have not yet been reported in people with type 2 diabetes in a longitudinal manner, that is, from measurements before and after COVID-19. Thus, this study aimed to assess the effect of COVID-19 in persons with type 2 diabetes on (a) HL content and adipose tissue compartments using state-of-the-art methods and on (b) measures of insulin resistance and beta-cell function.

## Research design and methods

### Participants

All participants were recruited from the ongoing prospective German Diabetes Study (GDS), which prospectively investigates persons with recent-onset diabetes, aged 18–69 years.^8^ The study was registered at ClinicalTrials.gov (NCT01055093). Also, the additional follow-up visit of the current study was approved by the local ethics committee. Informed consent was obtained from all participants prior to inclusion. Diabetes mellitus was classified according to current guidelines of the American Diabetes Association.^9^ Exclusion criteria comprised pregnancy, acute or severe chronic cardiac, renal or psychiatric diseases, known acute or severe chronic liver disease (clinical signs or transaminases at twofold above upper limit of the reference range), immunosuppressive treatment and acute inflammation (high-sensitivity C reactive protein >1 mg/dL).^10^

**Figure 1:**
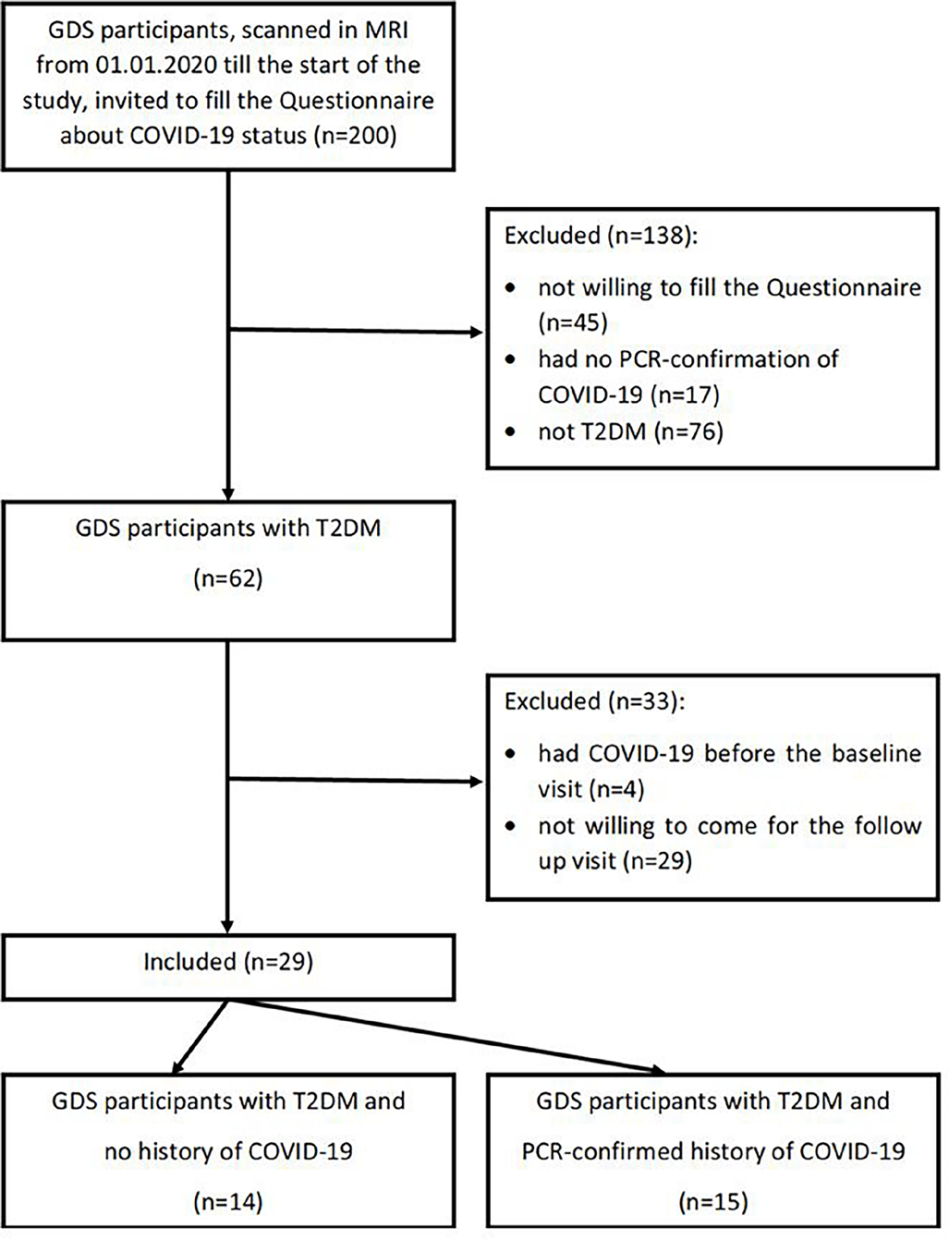
Recruitment flow diagram. GDS, German Diabetes Study; T2DM, type 2 diabetes mellitus.

Participants with type 2 diabetes, who had their last regular examination within GDS shortly before or shortly after the start of the SARS-CoV-2 pandemic in Germany (baseline visit for the current study), and had no COVID-19 before this examination were recruited for the additional follow-up visit. This resulted in 29 participants with type 2 diabetes, of whom 14 persons had no COVID-19 (COVID-neg group) and 15 persons had SARS-CoV-2 infection confirmed (PCR confirmed (COVID-pos group)) before the follow-up visit (figure 1).

### Multiparametric MR measurements

All MR measurements were performed after overnight fasting on a clinical 3-T MR scanner (Achieva X-series, Philips Healthcare, The Netherlands).

Whole-body fat (WBF), subcutaneous adipose tissue (SAT) and VAT volumes were quantified by whole-body MRI.^11^ Liver volume was determined from dual-echo Dixon measurements. Hepatic proton density fat fraction, as the MRI-based HL content measure (HL-PDFF), and T_2_* maps, as a measure of hepatic iron content, were determined from mDixon-Quant measurements, sampled at multiple sites throughout the liver.^12^ MASLD was defined by HL-PDFF ≥5%. Liver stiffness was determined using 2D gradient-echo MR elastography (MRE).^13^ To assess hepatic energy metabolism, absolute hepatic γATP and inorganic phosphate (Pi) concentrations were measured with ^31^P MR spectroscopy (MRS) using 3D image selected spectroscopy with ^1^H-decoupling.^11^ For quantification of intramyocellular lipids (IMCL), ^1^H MRS was performed in the tibialis anterior muscle.^14^ Details of the MRI, MRS, and MRE protocols are provided in the online supplemental material.

### Laboratory analyses

Routine laboratory parameters were analyzed in the biomedical laboratory.^8^ Homeostatic model assessment of insulin resistance (HOMA-IR) and beta-cell function (HOMA-B), triglyceride-glucose index, fatty liver index (FLI), fibrosis-4 index (FIB-4), aspartate aminotransferase to platelet ratio index (APRI), non-alcoholic fatty liver disease (NAFLD) score and Forns index were computed from routine laboratory parameters as described.^15^,^18^

### Statistics

Comparison between groups at baseline was performed using t-test (Fisher’s test for sex differences). Changes from the baseline to the follow-up visit, corrected for the time course in the absence of COVID-19, for log-normally distributed data are presented as relative changes with corresponding 95% CIs adjusted for changes in body mass index (BMI), the time interval between the baseline and the follow-up visits, and respective baseline parameter. Comparison of changes between COVID-neg and COVID-pos groups was done by an analysis of covariance adjusted for changes in BMI, the time interval between the baseline and the follow-up visits, and the baseline value of the respective parameter. P values ≤0.05 were considered to indicate statistically significant differences. In absence of data on metabolic alterations of hepatic or adipose tissue after recovery from COVID-19, sample size was based on Cohen’s calculation in order to assess the minimal number of participants. Assuming an effect slightly larger than the SD of measured parameters and setting Cohen’s d to 1.2 yield a sample size of 12 participants per group. Of note, the present study is not a preplanned intervention trial but an exploratory pilot study. All statistical analyses were performed with SAS (V.9.3; SAS Institute).

We used the Strengthening the Reporting of Observational Studies in Epidemiology cross-sectional checklist when writing our report.^19^

## Results

### Baseline characteristics

Anthropometric data of both groups were comparable except for nominally higher percentage of females and higher high-density lipoprotein-cholesterol in the COVID-neg group (table 1).

**Table 1:** Participants’ characteristics at baseline

Parameter	COVID-neg	COVID-pos
Number (n; % females)	14 (43)	15 (20)
Age (years)	61±6	58±12
Known diabetes duration (years)	7.5±3.6	7.7±4.6
Time interval between baseline and follow-up visits (months)	22±8	25±9
BMI (kg/m^2^)	30.0±4.6	30.9±5.2
Fasting blood glucose (mg/dL)	160±48	150±40
HbA1c (%) (mmol/mol)	7.1±1.0, 55±11	6.8±1.0, 51±11
HOMA-IR (au)	105 (46, 199)	119 (88, 176)
HOMA-B (au)	1.2 (0.6, 1.8)	2.1 (1.4, 3.2)
TyG (au)	19 022 (15 375, 34 030)	21 268 (14 212, 53 255)
hsCRP (mg/dL)	0.3±0.3	0.2±0.2
Fasting total cholesterol (mg/dL)	191±37	165±39
Fasting LDL-cholesterol (mg/dL)	121±39	101±32
Fasting HDL-cholesterol (mg/dL)	56±14*	40±12
Fasting triglycerides (mg/dL)	119 (101, 171)	135 (83, 342)
Fasting NEFA (µmol/L)	532±164	489±222
ALT (U/L)	26 (17, 35)	28 (25, 42)
AST (U/L)	23 (18, 26)	22 (18, 27)
GGT (U/L)	33 (21, 61)	25 (19, 38)

Data are shown as absolute numbers, percentages, mean±standard deviationSD or median [(interquartile rangeIQR]). ALT, alanine aminotransferase; AST, aspartate aminotransferase; GGT, gamma-glutamyl transferase; HbA1c, glycated hemoglobin A1c; HDL, high-density lipoprotein; HOMA-B, homeostatic model assessment of β-cell function, HOMA-IR, homeostatic model assessment of insulin resistance, TyG, Triglyceride-Glucose Index; LDL, low-density lipoprotein; NEFA, non-esterified fatty acids. Unpaired t-test: *, pp<0.05.

ALTalanine aminotransferaseASTaspartate aminotransferaseBMIbody mass indexGGTgamma-glutamyl transferaseHbA1cglycated hemoglobin A1cHDLhigh-density lipoproteinHOMA-Bhomeostatic model assessment of beta-cell functionHOMA-IRhomeostatic model assessment of insulin resistancehsCRPhigh-sensitivity C reactive proteinLDLlow-density lipoproteinNEFAnon-esterified fatty acidTyGtriglyceride-glucose index

### Follow-up after COVID-19

None of the participants of the COVID-pos group had severe COVID-19 according to the classification by the WHO, that is, none was hospitalized or had impaired pulmonary function defined by oxygenation <90% during the infection.^20^ The interval between PCR-confirmed COVID-19 diagnosis and follow-up visits was 12±2 months (range 7–14 months).

### Effect of COVID-19 on the liver

**Figure 2:**
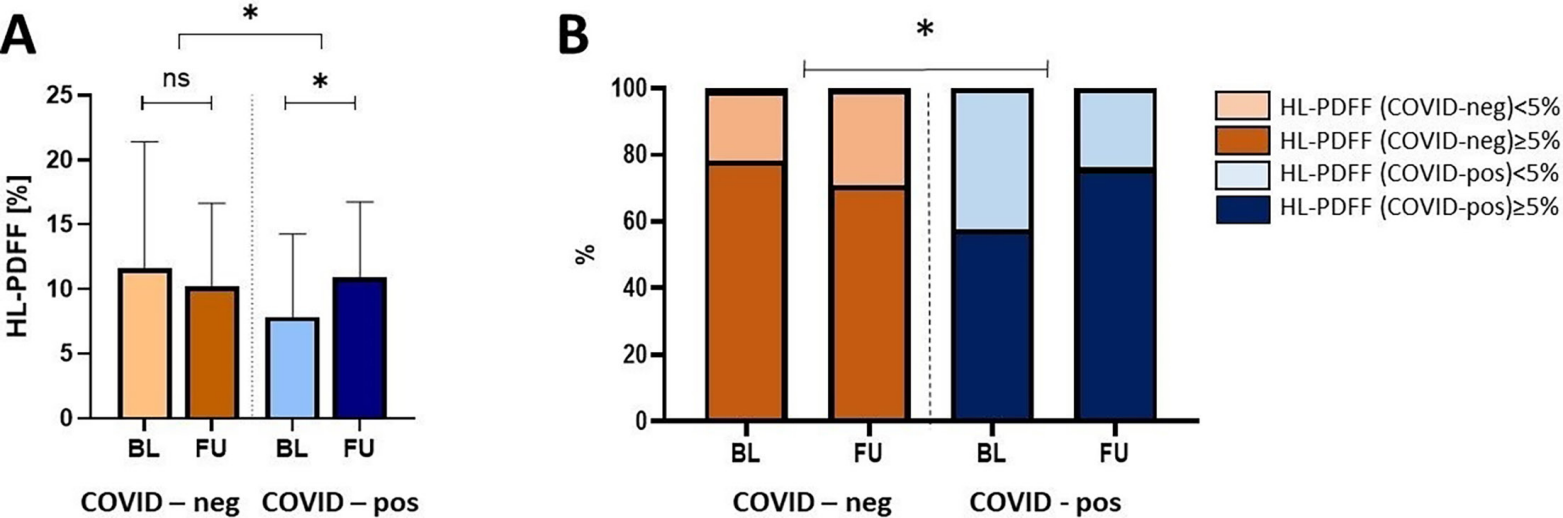
**(A**) Effect of history of COVID-19 on HL-PDFF. (**B**) Prevalence of metabolic dysfunction-associated steatotic liver disease (MASLD) in the studied groups based on HL-PDFF measurements. Unadjusted values of HL-PDFF are shown as mean±SD. *P≤0.05, indicating significance level for effect of COVID-19, corrected for the time course in the absence of COVID-19, based on analysis of covariance (ANCOVA) with adjustment for changes in body mass index (BMI), the time interval between the baseline and the follow-up visits, and the baseline value of the respective parameter. BL, baseline; FU, follow-up; HL-PDFF, hepatic lipid content measured as proton density fat fraction; ns, non-significant.

At the baseline visit, HL-PDFF values were comparable between both groups (COVID-neg 8% (95% CI 5%, 14%), COVID-pos 5% (95% CI 3%, 9%)). At follow-up, HL-PDFF slightly decreased in COVID-neg (relative decrease: −4%), but increased in COVID-pos (relative increase: +80%). Thus, COVID-19 resulted in a relative increase in HL-PDFF of 56% (95% CI 18%, 106%; p=0.04), corrected for the time course in the absence of COVID-19 (figure 2A). No such difference was found for the surrogate steatosis index FLI (2.3 (4.5, 9.1); p=0.24).

MRE-based hepatic stiffness (−0.11 kPa (−0.44, 0.23); p=0.43) as well as surrogate indices of liver fibrosis risk FIB-4 (−0.14 (−0.46, 0.19); p=0.10), APRI (−0.06 (−0.19, 0.06); p=0.14), NAFLD score (−0.52 (−0.89, –0.17); p=0.18) and Forns index (−0.10 (−0.54, 0.35); p=0.95) did not change in the COVID-pos group, when corrected for the time course in the absence of COVID-19. Similarly, no change in liver volume (−13 cm^3^ (−180, 154); p=0.22) and the surrogate of hepatic iron content, T_2_* (−0.41 ms (−1.94, 1.12); p=0.34), was detected for the COVID-pos group, when corrected for the time course in the absence of COVID-19.

Also, absolute concentrations of hepatic γATP and Pi remained unaffected by COVID-19 (ATP: 0.08 mmol/L (−0.87, 1.02); p=0.40 and Pi: −0.31 mmol/L (−0.99, 0.36); p=0.39, respectively).

To investigate the impact of MASLD at the baseline visit of the current study, the COVID-neg and COVID-pos groups were stratified into participants with and without MASLD, and the interaction term of COVID-19 history and MASLD status (yes/no) was added to the model. MASLD, as defined by HL-PDFF ≥5%, was present in 79% COVID-neg and in 58% COVID-pos participants at baseline. However, at the follow-up visit the proportion of participants with MASLD increased to 77% in the COVID-pos group, while it decreased in the COVID-neg group to 71%, and the interaction between a history of COVID-19 and MASLD status has reached statistical significance (p=0.05) (figure 2B).

### Effect of COVID-19 on insulin resistance and beta-cell function

HOMA-IR decreased in both groups from baseline to follow-up (relative reduction: COVID-neg –33%, COVID-pos –19%) to a similar extent; therefore, there was no effect of COVID-19, as there was no change in the COVID-pos group, when corrected for the time course in the absence of COVID-19 (−19% (−42, 13); p=0.49). HOMA-B similarly increased in both groups at follow-up (relative increase COVID-neg 8%, COVID-pos 4%); therefore, again, no changes in COVID-pos individuals were detected, when corrected for the time course in the absence of COVID-19 (4% (−25, 43); p=0.86).

### Effect of COVID-19 on adipose tissue compartments

COVID-19 did not result in changes when corrected for the time course in the absence of COVID-19 in WBF (−2 cm^3^ (−1814, 1809); p=0.21), SAT (148 cm^3^ (−1339, 1635); p=0.45) and VAT (61 cm^3^ (−695, 816); p=0.41) compartments. The same holds true for IMCL (−0.03 (−0.2, 0.1); p=0.47).

## Discussion and conclusion

Employing MR-based techniques, the present study demonstrates that even non-severe COVID-19 may exert a long-term effect on HL-PDFF in persons with type 2 diabetes, but neither on hepatic stiffness, volume and T_2_* nor on adipose tissue volumes.

Potential mechanisms leading to causing or worsening hepatic steatosis following COVID-19 include endoplasmic reticulum (ER) stress and abnormal hepatic mitochondrial function.^4^ Electron microscopic analysis showed ER dilation and mitochondrial swelling in postmortem liver biopsies of individuals with COVID-19.^21 22^ Alterations in mitochondrial function due to SARS-CoV-2 infection comprise downregulation of genes responsible for amino acid oxidation,^23 24^ higher levels of circulating non-esterified fatty acids,^25 26^ and upregulation of fatty acid biosynthesis.^27^ Of note, hepatic ER stress and abnormal mitochondrial function are known contributors to the development of MASLD.^28 29^ MASLD, in turn, is associated with a risk of type 2 diabetes,^30^ which is also elevated for up to 2 years following COVID-19.^31^

The present study benefits from using the currently most accurate non-invasive MR methods and the prospective design of the GDS, which allowed to evaluate measurements pre/post COVID-19 infection. This allows to investigate the effect of COVID-19 on organs beyond the respiratory system in a longitudinal way. To the best of our knowledge, this study is the first to show an effect of COVID-19 on HL content, measured using MRI, comparing data obtained before and after the disease in diabetes type 2 population.

Limitations of the study include the relatively small number of participants and the lack of information about possible lifestyle (physical activity, dietary habits) changes on COVID-19. On the other hand, the study cohort underwent comprehensive phenotyping, and changes in BMI between baseline and follow-up were monitored. As changes in body mass are main drivers of liver lipid content, we have adjusted all results for BMI. Finally, the glucose clamp remains the gold standard for measurement of insulin sensitivity, but the surrogate indices used in the current study, HOMA-IR and HOMA-B, have been extensively validated^32^ and yielded comparable results also in individuals with type 2 diabetes.^33^

Taken together, this study shows that people with type 2 diabetes have higher HL content at about 1 year after recovery from COVID-19 infection.

## supplementary material

10.1136/bmjdrc-2024-004727 online supplemental file 1

## Data Availability

Data are available upon reasonable request.
