# Supplementary material for: Alterations of hepatic lipid content following COVID-19 in persons with type 2 diabetes
Source: BMJ Open Diabetes Res Care. 2025 Feb 18;13(1):e004727. doi: 10.1136/bmjdrc-2024-004727 (PMC11836859; doi:10.1136/bmjdrc-2024-004727)
Supplement: online supplemental file 1 [file bmjdrc-13-1-s001.docx]

**Alterations of hepatic lipid content following COVID-19**

**in persons with type 2 diabetes**

**Short running title: Hepatic Lipids after COVID in Type 2 Diabetes**

Yuliya Kupriyanova, PhD*^1,2^, Iryna Yurchenko, MD*^1,2^, Pavel Bobrov, PhD^2,3^, Frederik Bartels, MSc^1,2^, Stefan Wierichs^1,2^, Marc Jonuscheit, MSc^1,2^, Benedict Korzekwa, MSc^1,2^, Katsiaryna Prystupa, MD^1,2^, Martin Schön, MD^1,2^, Dania Mendez, MD^1,2^, Sandra Trenkamp, PhD^1,2^, Volker Burkart, PhD^1,2^, Robert Wagner, MD^1,2,4^, Vera Schrauwen-Hinderling, PhD^1,2,5^, and Michael Roden, MD^4,1,2^ for the GDS group^#^

**Supplementary material**

**Summary of applied magnetic resonance based methods.**

Whole body fat (WBF), subcutaneous adipose tissue (SAT) and visceral adipose tissue (VAT) volumes were quantified by whole-body MRI by employing T1-weighted fast spin-echo (repetition time (TR) = 400 ms, echo time (TE) = 38 ms, 10 mm slice thickness with 10 mm gap between slices) (1) and post-processing by a trained operator using SliceOmatic® v5.0 software (Tomovision, Montréal, Canada).

Liver volume was determined from dual-echo Dixon measurements (TR = 3.76 ms, (TE) 1/2 = 1.32 / 2.40 ms, slice thickness = 2 mm, no gap) and processed using SliceOmatic® v5.0 software (Tomovision, Montréal, Canada).

Liver proton density fat fraction (PDFF) and T2* maps were determined from mDixon-Quant measurements (6-echo 3D Dixon: TR /TE / ΔTE = 5.8 / 1 / 0.7 ms, slice thickness = 3 mm, no gap). PDFF and T2* values were calculated by placing four regions of interest on the corresponding axial images at the portal vein level (2).

Liver stiffness was determined using a 2D gradient-echo – magnetic resonance elastography (MRE) (TR / TE = 50 / 20 ms, motion encoding gradient frequency = 60 Hz) (3). Four slices of 10 mm thickness were acquired in axial plane at the largest portion of the liver in coronal view, avoiding the heart, the liver dome, and the liver bottom tip. An MR elastogram was generated by using the MREview package (Philips, Best, Netherlands) and liver stiffness was calculated as described (4).

Absolute hepatic γATP and Pi concentrations were measured with 31P MRS using 3D image selected spectroscopy with 1H-decoupling (repetition time = 6000 ms, number of signal averages = 128) within a VOI of 60 ∗ 60 ∗ 60 mm^3^, as reported(5). The coefficient of variance of this 31P MRS method is <9 %, indicating high reliability for follow up studies (6).

For assessment of intramyocellular lipids (IMCL), water-suppressed and nonsuppressed 1H MRS was performed in a voxel (10 ∗ 10 ∗ 20 mm^3^) placed within the tibialis anterior muscle on the left leg using point resolved spectroscopy sequence (TR / TE = 2000 / 29 ms). IMCL content was calculated from the peak areas of IMCL-CH2 at 1.3 ppm with respect to the water peak area and was corrected for T1 and T2 relaxation effects, as reported previously (7).

**Supplementary references**

1. Machann J, Thamer C, Stefan N, Schwenzer NF, Kantartzis K, Haring HU, Claussen CD, Fritsche A, Schick F. Follow-up whole-body assessment of adipose tissue compartments during a lifestyle intervention in a large cohort at increased risk for type 2 diabetes. Radiology 2010;257:353-363

2. Campo CA, Hernando D, Schubert T, Bookwalter CA, Pay AJV, Reeder SB. Standardized Approach for ROI-Based Measurements of Proton Density Fat Fraction and R2* in the Liver. AJR Am J Roentgenol 2017;209:592-603

3. Loomba R, Wolfson T, Ang B, Hooker J, Behling C, Peterson M, Valasek M, Lin G, Brenner D, Gamst A, Ehman R, Sirlin C. Magnetic resonance elastography predicts advanced fibrosis in patients with nonalcoholic fatty liver disease: a prospective study. Hepatology (Baltimore, Md) 2014;60:1920-1928

4. Pepin KM, Welle CL, Guglielmo FF, Dillman JR, Venkatesh SK. Magnetic resonance elastography of the liver: everything you need to know to get started. Abdom Radiol (NY) 2022;47:94-114

5. Kupriyanova Y, Zaharia OP, Bobrov P, Karusheva Y, Burkart V, Szendroedi J, Hwang JH, Roden M. Early changes in hepatic energy metabolism and lipid content in recent-onset type 1 and 2 diabetes mellitus. J Hepatol 2021;74:1028-1037

6. Jonuscheit M, Wierichs S, Rothe M, Korzekwa B, Mevenkamp J, Bobrov P, Kupriyanova Y, Roden M, Schrauwen-Hinderling VB. Reproducibility of absolute quantification of adenosine triphosphate and inorganic phosphate in the liver with localized (31) P-magnetic resonance spectroscopy at 3-T using different coils. NMR Biomed 2024:e5120

7. Krssák M, Mlynárik V, Meyerspeer M, Moser E, Roden M. 1H NMR relaxation times of skeletal muscle metabolites at 3 T. Magma 2004;16:155-159
